# Genomic Characterization of an Echovirus 7 Isolate of Southeast Asian Ancestry Recovered from a Child in Nigeria with Acute Flaccid Paralysis

**DOI:** 10.1128/MRA.00830-19

**Published:** 2019-08-15

**Authors:** T. O. C. Faleye, O. M. Adewumi, J. A. Adeniji

**Affiliations:** a Department of Virology, College of Medicine, Faculty of Basic Medical Sciences, University of Ibadan, Ibadan, Nigeria; b Center for Human Virology and Genomics, Microbiology Department, Nigerian Institute of Medical Research, Lagos, Nigeria; c WHO National Polio Laboratory, University of Ibadan, Ibadan, Nigeria; Queens College

## Abstract

Here, we describe the genome of an echovirus 7 (E7) isolate of Southeast Asian ancestry recovered from a child in Nigeria with acute flaccid paralysis (AFP). The genome has 7,295 nucleotides (nt) and an open reading frame (ORF) with 2,195 amino acids.

## ANNOUNCEMENT

Echovirus 7 (E7) belongs to species B within the genus Enterovirus, family Picornaviridae, order Picornavirales (http://www.picornaviridae.com/enterovirus/enterovirus.htm), and it has been associated with various clinical manifestations (1). In 2014, we detected an E7 isolate from the feces of a child with acute flaccid paralysis (AFP) in Nigeria (2). The isolate, however, seemed to be of Southeast Asian ancestry (2). Here, we describe the full genome sequence of this isolate.

For this study, the isolate was recovered from storage at −20°C and passaged twice in an RD cell line culture (3). Subsequently, genomic RNA was extracted using a total RNA extraction kit (Jena Bioscience, Jena, Germany) and converted to cDNA using the Script cDNA synthesis kit (Jena Bioscience) in accordance with the manufacturer’s instructions. Amplification of the genome was done in overlapping fragments as previously described (4); fragments were pooled and shipped to a commercial sequencing facility (MR DNA, TX, USA), where library preparation (using the Nextera DNA sample preparation kit [Illumina]) and paired-end Illumina sequencing (300 cycles using the HiSeq system) were done.

Quality checking and *de novo* assembly were done using FastQC v1.0.4 and the Kiki assembler v0.0.9, respectively, with default parameters. The E7 genome is 7,295 nucleotides (nt) (G+C content of 48.1%) long and was assembled from 3,073,179 (93.87%) of the 3,273,740 reads generated, with a coverage of 62,187×. It has an open reading frame with 2,195 amino acids. A BLASTn search of the GenBank database showed the genome to be most similar (83.64%) to strain 40/Longyou/ZJ (GenBank accession number KU355273), which was recovered from cerebrospinal fluid from a case of viral encephalitis in Zhejiang, China (unpublished data). The same search done with only the VP1 region showed it to be most similar (91.48%) to strain N-311 (GenBank accession number JN203720), which was recovered from the stool of a child with AFP in India (5). This confirmed our previous findings of its Southeast Asian ancestry and its association with neurological manifestations (2).

When an E7 isolate recovered from sewage-contaminated water in Nigeria in 2010 (strain_Ibadan_NGR_2010, GenBank accession number MH732737) (2) was compared using MEGA5 (6) with the strain recovered in this study, the complete open reading frame (ORF), P1, and VP1 were 79.7%, 75.9%, and 76.5% similar at the nucleotide level, respectively. At the amino acid level, they were 96.6%, 95.2%, and 94.7% similar, respectively. When the similarities between the E7 genome described here and strain_Ibadan_NGR_2010 (MH732737) were reestimated with the exclusion of the wobble positions (third nucleotide) of all codons, the values for the similarities at the nucleotide level were reduced to 96.8%, 96.1%, and 95.2% for the ORF, P1, and VP1 genomic regions, respectively. The similarities between these and the amino acid values suggest that the divergence between the similarities at the nucleotide and amino acid levels could be almost entirely accounted for by codon bias.

We describe here an E7 genome, recovered from a child in Nigeria with AFP, that is of Southeast Asian ancestry and belongs to a lineage associated with neurological manifestations. More genomes from this lineage might provide the information needed to figure out the basis of their predilection for the central nervous system (CNS).

### Data availability.

The genome described has been deposited in GenBank and SRA under the accession numbers MK159694 and PRJNA505103, respectively.
